# Prevalence of measles antibodies among migrant workers in Singapore: a serological study to identify susceptible population subgroups

**DOI:** 10.1186/s12879-022-07066-2

**Published:** 2022-01-25

**Authors:** Li Wei Ang, Qi Gao, Lin Cui, Aysha Farwin, Matthias Paul Han Sim Toh, Irving Charles Boudville, Mark I-Cheng Chen, Angela Chow, Raymond Tzer-Pin Lin, Vernon Jian Ming Lee, Yee Sin Leo

**Affiliations:** 1 National Public Health and Epidemiology Unit, National Centre for Infectious Diseases, 16 Jalan Tan Tock Seng, Singapore, 308442 Singapore; 2 Public Health Group, Ministry of Health, Singapore, Singapore; 3 National Public Health Laboratory, National Centre for Infectious Diseases, Singapore, Singapore; 4 Saw Swee Hock School of Public Health, National University of Singapore, Singapore, Singapore; 5 Lee Kong Chian School of Medicine, Nanyang Technological University, Singapore, Singapore; 6 Department of Clinical Epidemiology, Office of Clinical Epidemiology, Analytics, and Knowledge (OCEAN), Tan Tock Seng Hospital, Singapore, Singapore; 7 Yong Loo Lin School of Medicine, National University of Singapore, Singapore, Singapore; 8 Department of Laboratory Medicine, National University Hospital, National University Health System, Singapore, Singapore; 9 National Centre for Infectious Diseases, Singapore, Singapore; 10 Department of Infectious Diseases, Tan Tock Seng Hospital, Singapore, Singapore

**Keywords:** Measles, Immunity, Prevalence, Vaccination coverage, Migrant workers

## Abstract

**Background:**

In 2019, two clusters of measles cases were reported in migrant worker dormitories in Singapore. We conducted a seroprevalence study to measure the level of susceptibility to measles among migrant workers in Singapore.

**Methods:**

Our study involved residual sera of migrant workers from seven Asian countries (Bangladesh, China, India, Indonesia, Malaysia, Myanmar and the Philippines) who had participated in a survey between 2016 and 2019. Immunoglobulin G (IgG) antibody levels were first measured using a commercial enzyme-linked immunosorbent assay (ELISA) test kit. Those with equivocal or negative IgG results were further evaluated using plaque reduction neutralization test (PRNT).

**Results:**

A total of 2234 migrant workers aged 20–49 years were included in the study. The overall prevalence of measles IgG antibodies among migrant workers from the seven Asian countries was 90.5% (95% confidence interval 89.2–91.6%). The country-specific seroprevalence ranged from 80.3 to 94.0%. The seroprevalence was significantly higher among migrant workers born in 1965–1989 than those born in 1990–1999 (95.3% vs. 86.6%, *p* < 0.0005), whereas there was no significant difference by gender (90.8% in men vs. 89.9% in women, *p* = 0.508). 195 out of 213 samples with equivocal or negative ELISA results were tested positive using PRNT.

**Conclusion:**

The IgG seroprevalence in migrant workers was below the herd immunity threshold of 95% for measles. Sporadic outbreaks may occur in susceptible individuals due to high transmissibility of measles virus. Seroprevalence surveys can help identify susceptible subgroups for vaccination.

**Supplementary Information:**

The online version contains supplementary material available at 10.1186/s12879-022-07066-2.

## Background

Measles is a highly contagious viral disease and poses a significant threat to public health. It is preventable by active vaccination. The measles vaccine is safe, effective and inexpensive and it has been in use since the 1960s. Under the Global Vaccine Action Plan, all World Health Organization (WHO) regions have set a measles elimination goal, but there is considerable variation in vaccination coverage by region and country [1]. The global resurgence of measles in 2018 and 2019 has highlighted the urgent need for renewed focus on measles vaccination efforts, particularly in population subgroups with suboptimal vaccination coverage [2].

The foreign workforce made up about a quarter of Singapore’s population of 5.69 million people in 2020, including 0.85 million on work permits who mainly engage in semi-skilled work [3]. The annual incidence of measles in Singapore ranged from 3 to 29 per million population during the 10-year period from 2009 to 2018 (Additional file 1: Fig. S1) [4]. Measles vaccination was made compulsory for children under the National Childhood Immunization Program in 1985 [5]. Since 2011, the measles, mumps and rubella (MMR) immunization schedule has consisted of the first dose at 12 months and the second dose at 15–18 months of age [6]. The two-dose MMR vaccination coverage ranged from 88 to 92% in Singapore residents at 2 years of age from 2013 to 2018 [7]. The high population immunity among Singapore residents is well demonstrated by serological surveys with measles prevalence of 98% in those aged 1–17 years in 2018 [8] and over 95% in adults in 2004 [9]. However, the immunity level against measles among non-residents in Singapore is unknown, particularly in vulnerable population groups who may require catch-up vaccination.

In 2019, two measles outbreaks involving six cases each were reported in two migrant worker dormitories in Singapore from 14 to 27 June and from 9 to 29 July, respectively [10]. One cluster comprised four cases from Bangladesh and two from India, and the other cluster comprised five cases from Bangladesh and one from India [10]. The median age of measles cases in each cluster was 30 years [10]. As their childhood vaccination and serology status was unknown, an added measure to minimise the risk of further transmission through ring vaccination was initiated for all close contacts of the measles cases who did not have proof of vaccination or immunity [10]. Such measles outbreak occurrences in densely populated dormitories stress the importance of profiling the prevalence of measles antibodies among migrant workers for an accurate assessment of their immunity status.

We conducted a serological study to assess the level of susceptibility to measles among migrant workers in Singapore. The findings will provide guidance for planning outbreak prevention and control programmes in the migrant worker population.

## Methods

### Seroprevalence survey

The measles seroprevalence study was conducted in accordance with the Infectious Diseases Act [11] which provides for the use of residual blood samples for the purpose of public health surveillance.

The study involved residual sera collected between 2016 and 2019 from a survey on latent tuberculosis among 3584 migrant workers in Singapore from eight Asian countries with large number of migrant workers in Singapore (Bangladesh, China, India, Indonesia, Malaysia, Myanmar, the Philippines and Vietnam). They were recruited from 27 locations around Singapore [12]. About 67% were recruited from clinics providing health screening services for migrant workers, 30% from migrant worker dormitories and 3% from recreation centres catering to migrant workers. The inclusion criteria were: (1) aged 20–50 years; (2) no previous employment in Singapore; (3) stayed in Singapore for less than a year.

Migrant workers who participated in the survey on latent tuberculosis and provided consent for their data and residual sera to be used for future research were eligible for inclusion in our study. For this serological study, the minimum sample size for each country of origin and birth cohort was determined based on published reports of measles seroprevalence in Bangladesh [13], China [14], India [15] and Malaysia [16] (Additional file 1: Table S1) with a confidence level of 95% and relative precision of 5%. For migrant workers from Indonesia, Myanmar and the Philippines, we assumed their measles antibody prevalence was at least 85% in each birth cohort, which gave a minimum sample size of 272 with a confidence level of 95% and relative precision of 5%. We used random sampling to select migrant workers from each country of origin and birth cohort if the number of available residual sera exceeded the minimum sample size required. We excluded migrant workers from Vietnam from the analysis as there were only 15 residual sera available in total.

### Measurement of measles antibodies

Testing of the residual sera was conducted by the National Public Health Laboratory, the national reference laboratory for measles in Singapore. Immunoglobulin G (IgG) antibody levels were measured using an enzyme-linked immunosorbent assay (ELISA), Enzygnost® Anti-Measles Virus/IgG (Siemens, Germany). Test results were reported in corrected optical density (∆OD) and classified into three categories according to the manufacturer’s instructions: negative (∆OD < 0.1), equivocal (0.1 ≤ ∆OD ≤ 0.2) or positive (∆OD > 0.2).

Plaque reduction neutralization test (PRNT) is known as the gold standard assay for evaluation of humoral immunity to measles [17, 18]. Samples with equivocal and negative ELISA results were further evaluated using PRNT. The assay used was adopted from a standardized laboratory protocol for measles PRNT by the WHO which was subsequently validated for use in clinical trials of aerosolized measles vaccines [19]. The measles PRNT titre was calculated from the dilution that reduced the number of plaques by 50%, and a value ≥ 8 was considered positive.

### Statistical analysis

We used the Wilson method [20] to compute the 95% confidence intervals (CI) for binomial proportions. The Mantel–Haenszel chi-square test for trend was used to evaluate the difference in seroprevalence across age groups. Fisher’s exact test was used to test for differences in seroprevalence by gender. All analyses were performed using SPSS version 24 (IBM, USA). All *p* values reported were 2-sided and statistical significance was taken as *p* < 0.05.

## Results

A total of 2234 migrant workers with residual sera were included in the study. Their mean age was 27.3 years (standard deviation 5.1, range 20–49). Among them, 30.4% were from India, 20.1% from Indonesia, 12.9% from Bangladesh, 12.7% from Myanmar and the remaining were from China, the Philippines and Malaysia (Table 1). All migrant workers from Bangladesh and more than 94% from China, India and Malaysia were men. All except one from Indonesia, 94.1% from the Philippines and 83.5% from Myanmar were women.

**Table 1:** Number of migrant workers included in study by country of origin, birth cohort and gender

	Country of origin						
	Bangladesh	China	India	Indonesia	Malaysia	Myanmar	Philippines
Total	289	193	679	450	152	284	187
Birth cohort							
1965–1984	31	72	101	137	18	24	64
1985–1989	63	67	171	104	24	48	64
1990–1994	136	43	205	209	64	207	59
1995–1999	59	11	202	0	46	5	0
Gender							
Male	289	182	668	1	143	47	11
Female	0	11	11	449	9	237	176

**Fig. 1:**
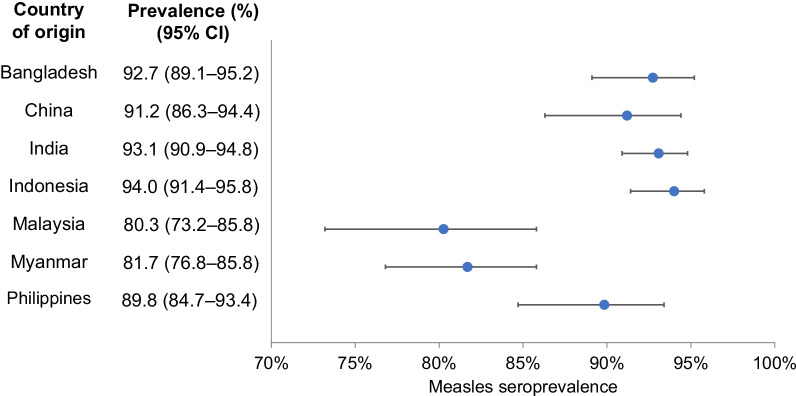
Measles IgG seroprevalence (%) measured by ELISA among migrant workers in Singapore by country of origin. The error bars indicate 95% confidence interval (CI)

The overall prevalence of measles IgG antibodies among the migrant workers from the seven Asian countries was 90.5% (95% confidence interval [CI] 89.2–91.6%). The seroprevalence was above 80% in migrant workers from each country of origin (Fig. 1). The seroprevalence ranged from 80.3% in workers from Malaysia to 94.0% from Indonesia. The country-specific seroprevalence was above 85% among workers born in 1965–1984 and those born in 1985–1989, and it exceeded 72% for both men and women (Table 2). When aggregated across the seven countries, the prevalence of measles IgG antibodies was significantly higher in migrant workers born in 1965–1989 than those born in 1990–1999 (95.3% vs. 86.6%, *p* < 0.0005), whereas there was no significant difference by gender (90.8% in men vs. 89.9% in women, *p* = 0.508).

**Table 2:** Measles IgG seroprevalence (%) measured by ELISA (95% confidence interval) among migrant workers in Singapore by country of origin, birth cohort and gender

	Country of origin						
	Bangladesh	China	India	Indonesia	Malaysia	Myanmar	Philippines
Birth cohort							
1965–1984	100 (89.0–100)	91.7 (83.0–96.1)	99.0 (94.6–99.8)	97.8 (93.8–99.3)	88.9 (67.2–96.9)	100 (86.2–100)	96.9 (89.3–99.1)
1985–1989	98.4 (91.5–99.7)	88.1 (78.2–93.8)	97.1 (93.3–98.7)	96.2 (90.5–98.5)	87.5 (69.0–95.7)	93.8 (83.2–97.9)	87.5 (77.2–93.5)
1990–1994	91.9 (86.1–95.4)	93.0 (81.4–97.6)	91.7 (87.1–94.8)	90.4 (85.7–93.7)	79.7 (68.3–87.7)	77.3 (71.1–82.5)	84.7 (73.5–91.8)
1995–1999	84.7 (73.5–91.8)	100 (74.1–100)	88.1 (82.9–91.9)	–	73.9 (59.7–84.4)	60.0 (23.1–88.2)	–
Gender							
Male	92.7 (89.1–95.2)	92.3 (87.5–95.4)	93.1 (90.9–94.8)	100 (20.7–100)	79.0 (71.6–84.9)	76.6 (62.8–86.4)	90.9 (62.3–98.4)
Female	–	72.7 (43.4–90.3)	90.9 (62.3–98.4)	94.0 (91.4–95.8)	100 (70.1–100)	82.7 (77.4–87.0)	89.8 (84.4–93.4)

There were 213 samples with equivocal or negative ELISA results. Among these, 195 (91.5%) tested positive using PRNT. Of 116 IgG-equivocal and 97 IgG-negative, 115 (99.1%) and 80 (82.5%) tested positive using PRNT, respectively.

## Discussion

In our serological study, the overall IgG seroprevalence of measles in migrant workers from all the seven Asian countries was below the herd immunity threshold of 95% for measles [21]. Sporadic measles outbreaks can occur in susceptible individuals, particularly in those originating from countries with suboptimal vaccination coverage. The high transmissibility of measles virus and close living environments of migrant worker dormitories may have led to the two clusters of measles cases reported in 2019 in Singapore. There have been reports of measles outbreaks associated with vaccine failure or low vaccine effectiveness in other countries [22–24].

In this study, the IgG seroprevalence was significantly higher among older migrant workers born in 1989 and earlier years. The age at vaccination is an important host factor in determining the immune response to measles-containing vaccine (MCV) [25]. Most studies on the immunogenicity of standard-titre MCV did not report on gender differences in seroconversion rates [25].

Measles is endemic in all the seven Asian countries where the migrant workers hailed from [26]. The humoral immunity against measles observed in migrant workers is more likely to be due to past infection for those born before or in the first few years when childhood measles vaccination was introduced in the countries where they hailed from. In 1995, the vaccination coverage of the first dose for measles in children aged ≤ 1 year ranged from 72% in India to 86% in Malaysia (Additional file 1: Table S2). More than two decades later, the measles vaccination coverage of the first dose in 2019 increased in all these countries except in the Philippines [27–34] (Additional file 1: Table S2).

Upon arrival in Singapore, all migrant workers have to undergo a pre-employment health examination, which includes review of medical history, physical examination and screening, and subsequent screening [35]. There is no vaccination policy against infectious diseases such as measles for migrant workers. The measles seroprevalence of migrant workers observed in our study could be due to a combination of those with vaccine-derived and naturally acquired immunity [10].

In 2018, Singapore was verified as having achieved measles elimination by the WHO [36, 37]. However, Singapore still faces the risk of measles importations from international travellers and migrant workers [36]. The measles outbreaks in 2019 have prompted an evaluation of the need for a catch-up vaccination program for all migrant workers without documentation of receipt of two doses of MMR. Given the highly contagious nature of measles virus, a multi-pronged approach remains essential in minimizing the risk of disease transmission. Atypical clinical presentations, especially among those who have immunity to measles [38–41], pose a challenge to early detection of cases. Hence it is important to remain vigilant, conduct thorough epidemiological investigation of suspected cases, obtain respiratory samples to perform polymerase chain reaction tests and genotyping for these cases, and implement prevention and control measures despite high seroprevalence of measles in migrant workers [10].

Approximately 82.5% of the 97 samples which had been tested IgG-negative and all except one of 116 samples tested IgG-equivocal using ELISA were found to be seropositive after subsequent PRNT. If positive PRNT results were included in the classification of results, the country-specific measles seropositivity of migrant workers would have ranged from 97.9 to 100% instead of from 80.3 to 94.0% based on ELISA alone (Additional file 1: Table S3). It is not known whether the antibody levels of individuals who tested ELISA negative but PRNT positive correlate with protection against measles, and this should be determined by long-term follow-up.

As this study was based on residual sera from a survey on latent tuberculosis, and there was no published data on distribution of migrant workers by country of origin and age group in Singapore, we were unable to assess whether the study sample was representative of the migrant workers at large. The small number of migrant workers in some age groups limited the comparison by birth cohort and led to wide confidence intervals of the seroprevalence estimates.

## Conclusions

The IgG seroprevalence in migrant workers from each of the seven Asian countries was below the herd immunity threshold of 95% for measles. This study underscores the importance of periodic seroprevalence surveys to measure the level of immunity against measles in migrant workers, so as to identify susceptible subgroups for active vaccination.

## Supplementary Information

**Additional file 1: Figure S1.** Annual incidence of reported measles cases in Singapore, 2000–2018. **Table S1.** Seroprevalence of measles IgG antibodies in Bangladesh, China, India and Malaysia. **Table S2.** Vaccination schedule and vaccination coverage for measles, and incidence per million population of measles cases in Bangladesh, China, India, Indonesia, Malaysia, Myanmar and the Philippines. **Table S3.** Number and percentage of samples tested positive based on ELISA alone and those based on ELISA or PRNT by country of origin, birth cohort and gender.

## Data Availability

The data that support the findings of this study are available from Qi Gao, National Public Health and Epidemiology Unit, but restrictions apply to the availability of the data, which was used under license for the current study, and so are not publicly available. Data are however available from Qi Gao upon reasonable request.
