# Supplementary material for: Prevalence of measles antibodies among migrant workers in Singapore: a serological study to identify susceptible population subgroups
Source: BMC Infect Dis. 2022 Jan 25;22:88. doi: 10.1186/s12879-022-07066-2 (PMC8787927; doi:10.1186/s12879-022-07066-2)
Supplement: Supplementary file 1 — Additional file 1: Figure S1. Annual incidence of reported measles cases in Singapore, 2000–2018. Table S1. Seroprevalence of measles IgG antibodies in Bangladesh, China, India and Malaysia. Table S2. Vaccination schedule and vaccination coverage for measles, and incidence per million population of measles cases in Bangladesh, China, India, Indonesia, Malaysia, Myanmar and the Philippines. Table S3. Number and percentage of samples tested positive based on ELISA alone and those based on ELISA or PRNT by country of origin, birth cohort and gender. [file 12879_2022_7066_MOESM1_ESM.pdf]

Prevalence of measles antibodies among migrant workers in Singapore: a serological study to identify susceptible population subgroups

Li Wei Ang, Qi Gao, Lin Cui, Aysha Farwin, Matthias Paul Han Sim Toh, Irving Charles Boudville, Mark I–Cheng Chen, Angela Chow, Raymond Tzer–Pin Lin, Vernon Jian Ming Lee, Yee Sin Leo

Fig. S1. Annual incidence of reported measles cases in Singapore, 2000–2018

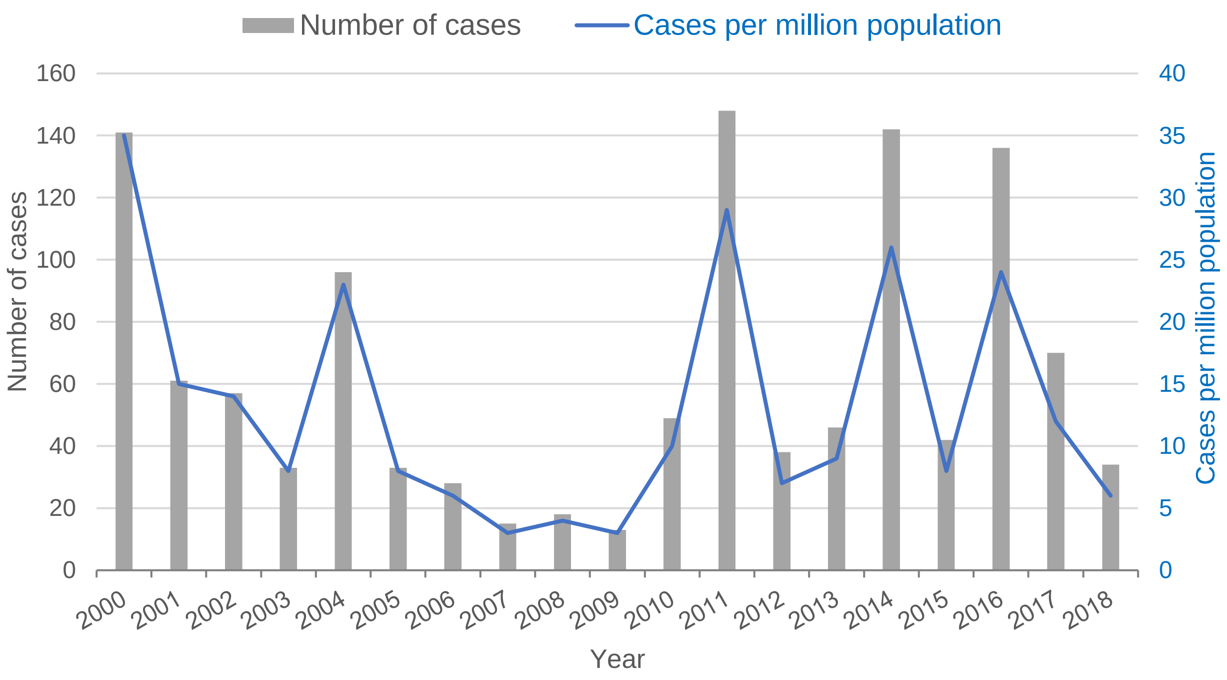

Table S1. Seroprevalence of measles IgG antibodies in Bangladesh, China, India and Malaysia

Country	Period of survey	Study group	Measles seroprevalence
Bangladesh [1]	2013	342 children aged 12–16 months in Mirzapur town	83.0%
China [2]	2016	3574 participants aged 2 months to 49 years in Shaanxi Province	85.9%
India [3]	2017–2018	335 adults (mean age: 20.5±1.4 years)	87.2%
Malaysia [4]	2014–2015	1541 participants aged 6–54 years in Seremban	87% in 6–54 year–olds,<br>90% in 6–9 year–olds,<br>74% in 15–24 year–olds,<br>94% in 45–54 year–olds

References:

[1] Hayford KT, Shomik MS, Al-Emran HM, Moss WJ, Bishai D, Levine OS. Measles vaccination coverage estimates from surveys, clinic records, and immune markers in oral fluid and blood: a population–based cross–sectional study. BMC Public Health. 2013;13:1211. doi: 10.1186/1471–2458–13–1211.

[2] Pei L, Yang Y, Zhao X, Zhang S, Yuan L, Liu Y, et al. Identify the susceptibility profile to measles in the general population: serological survey of measles antibodies in Shaanxi province, China, in 2016. Vaccine. 2017;35(52):7250–5. doi: 10.1016/j.vaccine.2017.11.012.

[3] Karade S, Sen S, Sashindran VK, Sharma P, Kanitkar M. Measles, mumps, and rubella: A cross-sectional study of susceptibility to vaccine–preventable diseases among young people in India. Med J Armed Forces India. 2019;75(1):70–3. doi: 10.1016/j.mjafi.2018.12.010.

[4] Hazlina Y, Marlindawati MA, Shamsuddin K. Serological assessment of the establishment of herd immunity against measles in a health district in Malaysia. BMC Infect Dis. 2016;16(1):740. doi: 10.1186/s12879–016–2069–y.

Table S2. Vaccination schedule and vaccination coverage for measles, and incidence per million population of measles cases in Bangladesh, China, India, Indonesia, Malaysia, Myanmar and the Philippines

Country	Year of introduction of childhood measles vaccination		Age recommended (months) for vaccination		Vaccination coverage			Incidence per million population in 2010–2019	
					Year 1995	Year 2019		Average annual rate	Highest annual rate
	1st dose	2nd dose	1st dose	2nd dose	1st dose	1st dose	2nd dose		
Bangladesh	1989	2012	9.5 (38 weeks)	15	79%	93%	89%	14.2	37.7
China	1978	1986	8	18	80%	99%	98%	15.7	38.6
India	1985	2010	9–12	16–24	72%	95%	84%	14.9	26.9
Indonesia	1985	2004	9, 24	84	82%	88%	71%	46.0	89.3
Malaysia	1985	2005	MMR: 9 months for children born July 2015 onwards Measles: 6 months, Sabah State only	MMR: 12 months for children born July 2015 onwards; MR: 84 months as second dose MCV for children born before July 2015.	86%	97%	87%	38.2	64.3
Myanmar	1990	2008	9	18	82%	84%	80%	26.0	97.2
Philippines	1985	2009	9–11	12–15	83%	73%	68%	144.7	585.5

MCV, measles containing vaccine, MMR, measles–mumps–rubella; MR, measles–rubella

Data sources:

World Health Organization. WHO vaccine–preventable diseases: monitoring system. 2020 global summary. Last updated 15 July 2020. Available at https://apps.who.int/immunization_monitoring/globalsummary. Accessed 3 April 2021.

World Health Organization. Measles reported cases. Last update: 15–Oct–2020 (data received as of 12–Oct–20). https://apps.who.int/immunization_monitoring/globalsummary/timeseries/tsincidencediphtheria.html. Accessed 9 April 2021.

Table S3. Number and percentage of samples tested positive based on ELISA alone and those based on ELISA or PRNT by country of origin, birth cohort and gender

	No. of samples	ELISA positive	ELISA or PRNT positive^
		No. (%)	No. (%)
**All**	2234	2021 (90.5)	2216 (99.2)
**Country of origin**			
Bangladesh	289	268 (92.7)	285 (98.6)
China	193	176 (91.2)	193 (100.0)
India	679	632 (93.1)	677 (99.7)
Indonesia	450	423 (94.0)	446 (99.1)
Malaysia	152	122 (80.3)	150 (98.7)
Myanmar	284	232 (81.7)	278 (97.9)
Philippines	187	168 (89.8)	187 (100.0)
**Birth cohort**			
1965–1984	447	433 (96.9)	445 (99.6)
1985–1989	541	509 (94.1)	538 (99.4)
1990–1994	923	803 (87.0)	912 (98.8)
1995–1999	323	276 (85.4)	321 (99.4)
**Gender**			
Male	1341	1218 (90.8)	1333 (99.4)
Female	893	803 (89.9)	883 (98.9)

ELISA, enzyme-linked immunosorbent assay; PRNT, plaque reduction neutralization test

^ This group comprised 2021 samples tested positive based on ELISA alone, and 195 samples tested equivocal or negative based on ELISA and subsequently tested positive using PRNT.
